# Supplementary figures and images for: Alterations of the Sympathoadrenal Axis Related to the Development of Alzheimer’s Disease in the 3xTg Mouse Model
Source: Biology (Basel). 2022 Mar 26;11(4):511. doi: 10.3390/biology11040511 (PMC9027376; doi:10.3390/biology11040511)

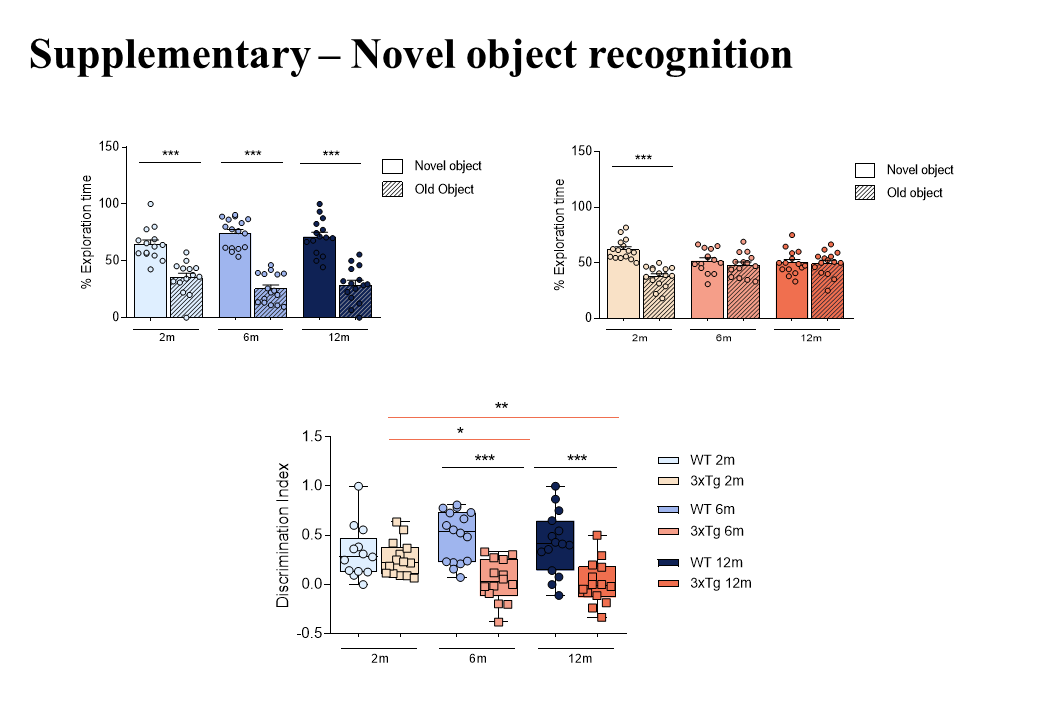

Supplement: Supplementary file 1 [file biology-11-00511-s001.zip › biology-1619535-supplementary.tif]
